# Editorial Note: Cardiac Fibroblast-Dependent Extracellular Matrix Accumulation Is Associated with Diastolic Stiffness in Type 2 Diabetes

**DOI:** 10.1371/journal.pone.0319058

**Published:** 2025-02-06

**Authors:** The PLOS One Editors

Following publication of this article [1], a concern was raised that the Ponceau staining loading control panels in Figs 2a, 2c, and 4b appear similar when the 4b image is flipped horizontally. The Materials and Methods section of the original article indicates that for analysis of protein expression in conditioned media, western blot membranes were stained with Ponceau Red and scanned images of Ponceau Red stained blots were used to correct for loading using an arbitrary band at 55–60 kDa. Therefore, when blots are stripped and re-probed for different proteins, the loading control bands are expected to be identical. No records are available to confirm that the blots in the published figures were stripped and re-probed, and the original underlying images and quantitative data are not available. However, a set of replicate blots from the original study and blots from a repeat experiment performed at a later date have been provided (S1 File) in which blots were stripped and re-probed for the different proteins.

The *PLOS One* Editors consider that the methodology for these experiments likely explains the appearance of duplicate loading controls in Figs 2a, 2c, and 4b; however, in view of the horizontally flipped orientation of one of the loading control panels, there is a discrepancy in the lane labels which cannot be fully resolved in the absence of the original underlying data. The corresponding author has stated that the loading control values used for the quantitative analysis were correct and not affected by the orientation error in the figures.

The *PLOS One* Editors further note the following limitations related to the replicate and replacement blots (S1 File): no quantitative data are available for these blots, and there is a high level of non-specific binding and poor visibility of some of the target protein bands. Readers are informed of these limitations so that the data may be interpreted accordingly.

## Supporting information

S1 File Replicate blots from the original study and repeat blots carried out in 2017. Replicate blots (right-hand images) and later repeat blots (left-hand images). Page 1: Coomassie stain; Page 2: PAI-1 immunoblot (equivalent to Fig 2c); Page 3: Collagen I immunoblot (equivalent to Fig 2a; note a different antibody is used in the later repeat experiment); Page 4: TIMP-2 immunoblot (equivalent to Fig 4b). Arrows indicate the molecular weight for the target protein bands. Sample labels differ between the S1 File and the original published article; correlative labels are as follows: Het 24 LG = *db/wt* NG or non-diabetic cells treated with normal glucose media (NG; low glucose, LG) for 24 hours; Het 24 LG>HG = *db/wt* HG or non-diabetic cells treated with hyperglycemic (high glucose, HG) media for 24 hours; Db 24 HG = *db/db* HG or diabetic cells treated with hyperglycemic (high glucose, HG) for 24 hours; Db 24 HG>LG = *db/db* NG or diabetic cells treated with normal glucose media (NG; low glucose, LG) for 24 hours.
(PDF)
