# Supplementary material for: Editorial Note: Cardiac Fibroblast-Dependent Extracellular Matrix Accumulation Is Associated with Diastolic Stiffness in Type 2 Diabetes
Source: PLoS One. 2025 Feb 6;20(2):e0319058. doi: 10.1371/journal.pone.0319058 (PMC11801573; doi:10.1371/journal.pone.0319058)

Flip Flop Glucose Gel
8.23.17

Gel E
10% Gel

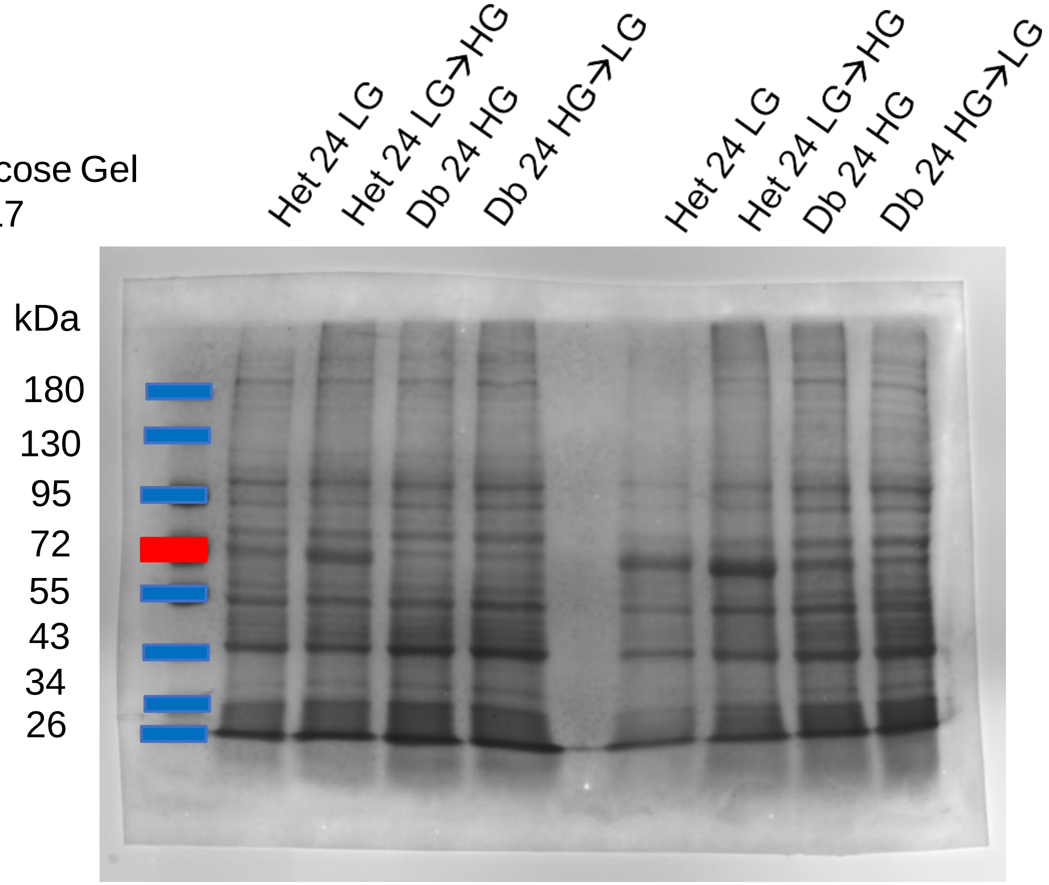

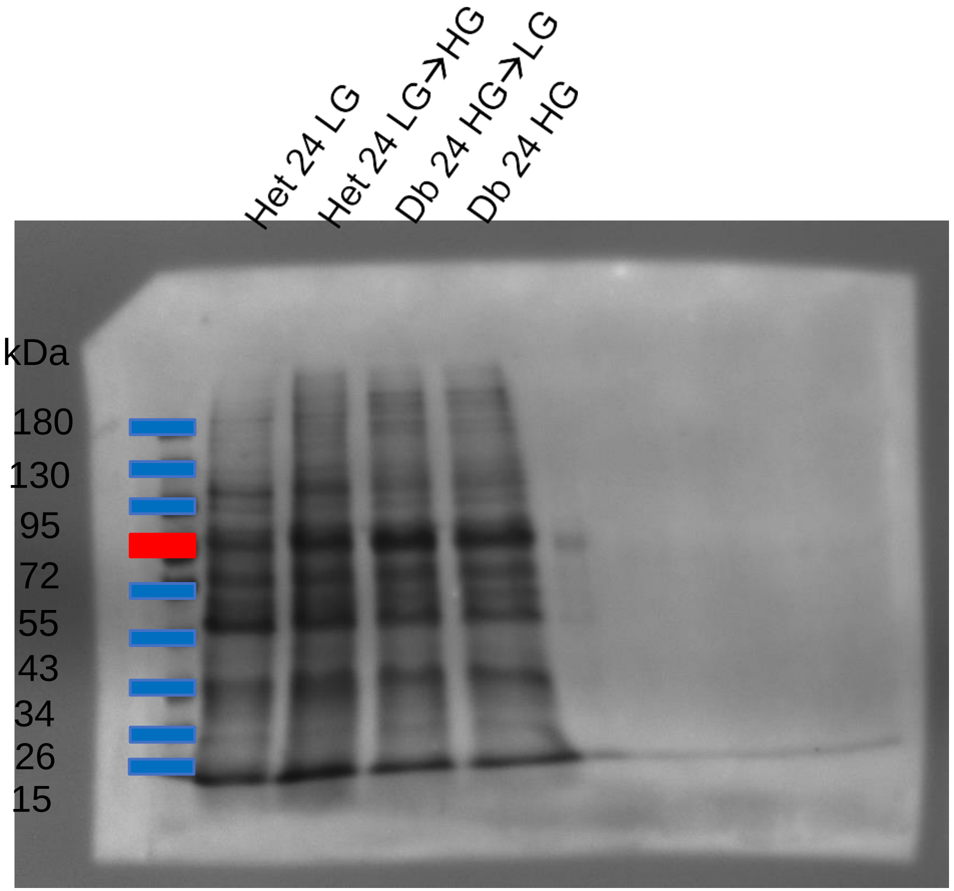

Flip Flop Glucose Gel
8.21.17
PAI-1; 50 kDa

Gel D
10% Gel

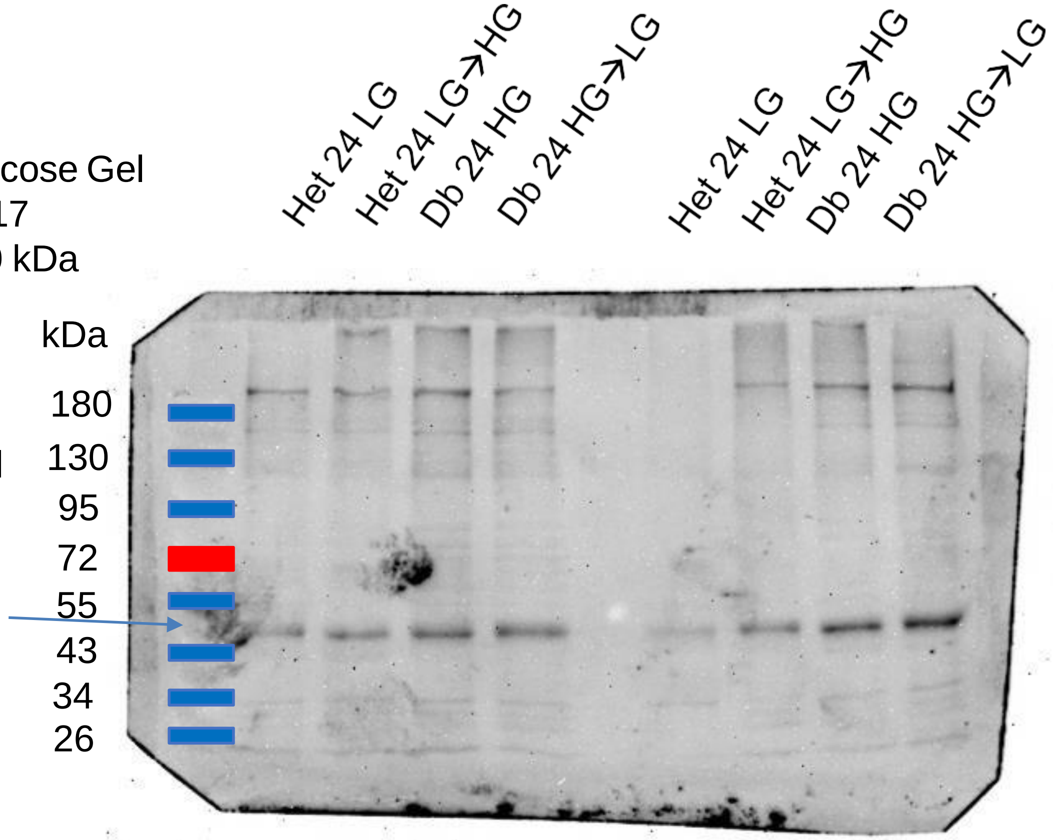

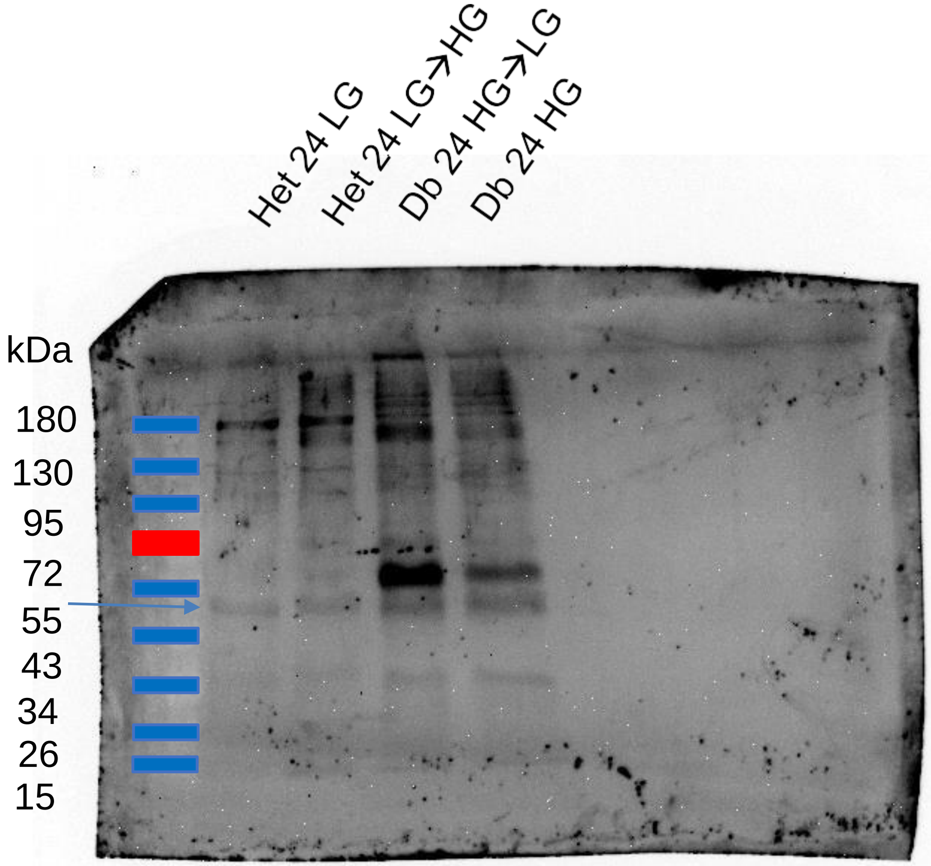

Flip Flop Glucose Gel
8.26.17
Collagen I; 240 kDa

Gel E
10% Gel

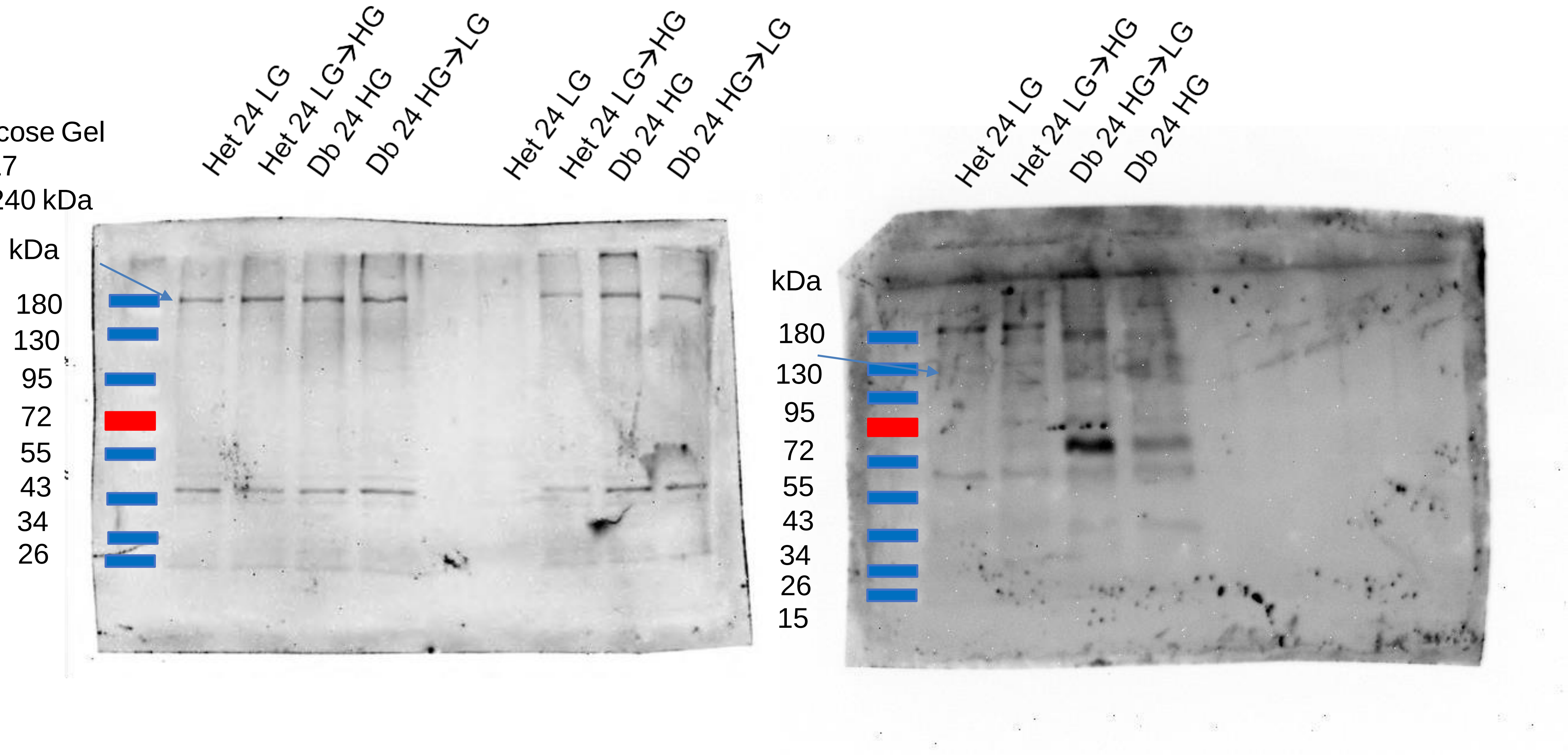

Flip Flop Glucose Gel
8.29.17
TIMP-2; 21 kDa

Gel E
10% Gel

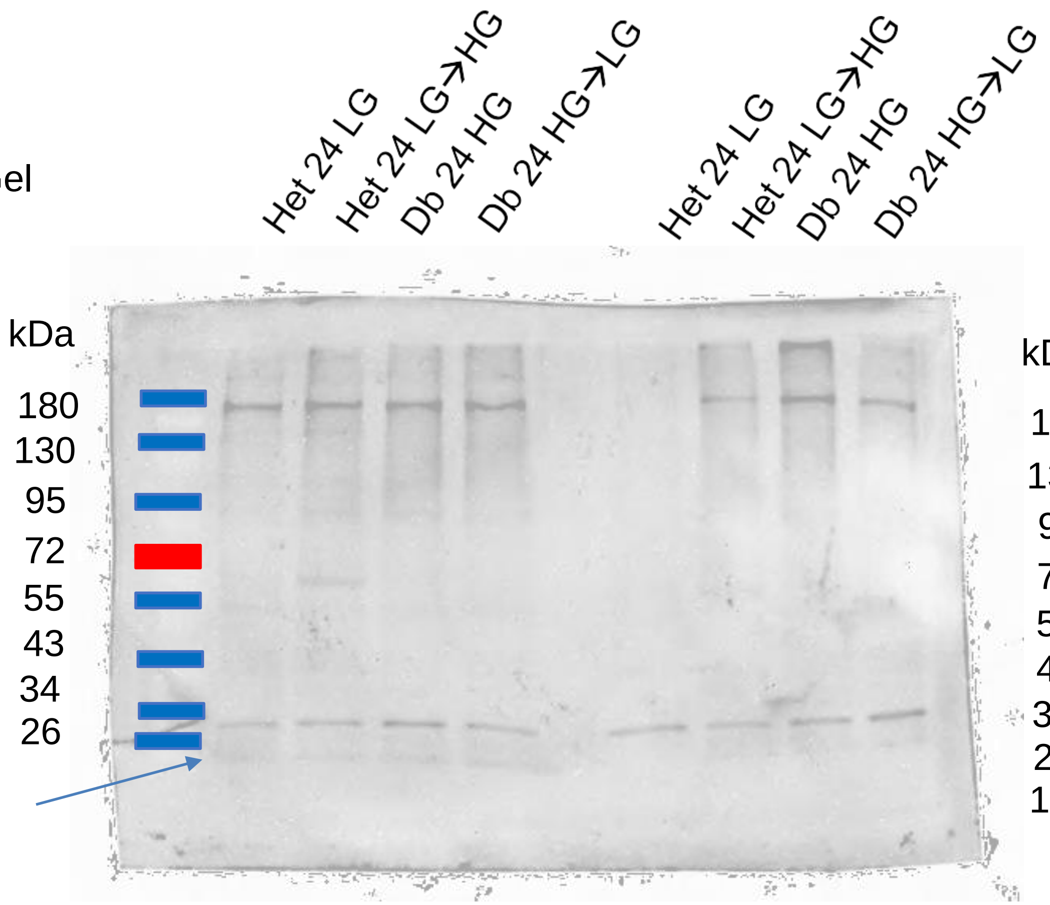

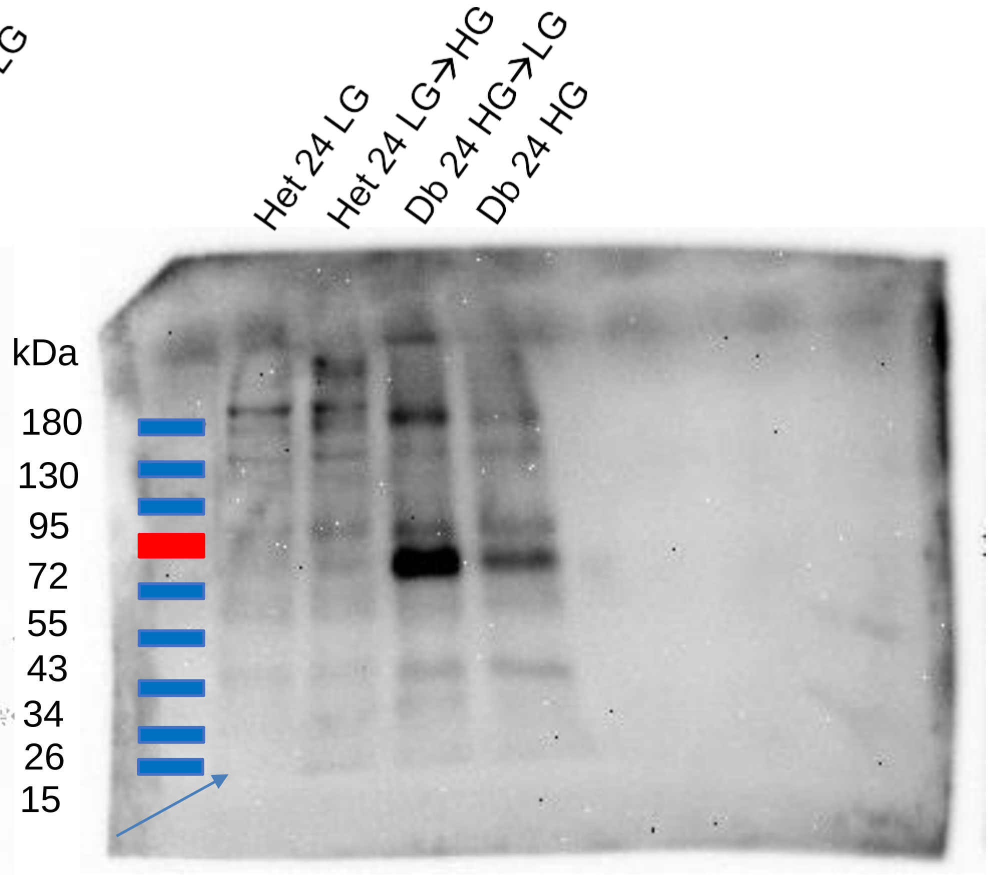

Supplement: S1 File — Replicate blots (right-hand images) and later repeat blots (left-hand images). Page 1: Coomassie stain; Page 2: PAI-1 immunoblot (equivalent to Fig 2c); Page 3: Collagen I immunoblot (equivalent to Fig 2a; note a different antibody is used in the later repeat experiment); Page 4: TIMP-2 immunoblot (equivalent to Fig 4b). Arrows indicate the molecular weight for the target protein bands. Sample labels differ between the S1 File and the original published article; correlative labels are as follows: Het 24 LG = db/wt NG or non-diabetic cells treated with normal glucose media (NG; low glucose, LG) for 24 hours; Het 24 LG>HG = db/wt HG or non-diabetic cells treated with hyperglycemic (high glucose, HG) media for 24 hours; Db 24 HG = db/db HG or diabetic cells treated with hyperglycemic (high glucose, HG) for 24 hours; Db 24 HG>LG = db/db NG or diabetic cells treated with normal glucose media (NG; low glucose, LG) for 24 hours. (PDF) [file pone.0319058.s001.pdf]
